# Spatial effects of the synergistic development between agricultural carbon sequestration and emission reduction and food security across China’s grain functional areas

**DOI:** 10.1186/s13021-025-00369-2

**Published:** 2025-12-12

**Authors:** Yidi Wang, Xianzhao Liu, Jiaxi Liu

**Affiliations:** 1 School of Earth Science and Spatial Information Engineering, Hunan University of Science and Technology, Xiangtan, 411201 China; 2 School of Economics, Guangxi Minzu University, Nanning, 530006 China

**Keywords:** Grain functional areas, Carbon sequestration and emission reduction, Food security, Coupled synergy, Spatial durbin model

## Abstract

**Background:**

Understanding the spatial effects of the synergistic development between agricultural carbon sequestration and emission reduction (ACSER) and food security (FS) is essential for promoting sustainable and high-quality agrarian development. Based on a constructed indicator system for ACSER and FS, this study measures the Coupling Coordination Degree (CCD) between the two in different grain functional areas of China from 2000 to 2023 using a modified coupling synergy model. Furthermore, a Spatial Durbin Model (SDM) is employed to explore the underlying driving mechanisms and spatial spillover effects of their synergistic relationship.

**Results:**

(1) From 2000 to 2023, China’s CCD between ACSER and FS exhibited a clear spatial gradient, higher in the north and lower in the south. Specifically, spatial clustering intensified in the main production and production-marketing areas but did not reach statistical significance, while the main marketing regions consistently exhibited a trend of spatial dispersion; (2) The spatial Durbin model analysis reveals that the CCD of ACSER and FS in China exhibits significant spatial spillover effects. Among the influencing factors, CO₂ uptake by major crops emerges as the primary driver of their synergistic development, while a higher proportion of cultivated land and increased pesticide and fertilizer use exert negative effects on both local and neighboring regions. (3)The study of regional heterogeneity shows that the CO₂ absorption of agricultural crops in the main production area promotes synergistic development; the synergistic mechanism of ACSER and FS in the production and marketing areas is more complicated, and resource mismatch is the main factor affecting the coupling and synergism of the two; The total power of agricultural machinery in the main marketing areas has a catalytic effect on the local synergy between ACSER and FS, while transregional transmission exhibits negative spillovers, highlighting resource allocation imbalances.

**Conclusions:**

Therefore, to promote the synergistic development of ACSER and FS, it is necessary to adopt region-specific measures based on spatial differences and to strengthen the synergistic effects among key factors within the agricultural system, to enhance resource allocation efficiency and system resilience.

**Supplementary Information:**

The online version contains supplementary material available at 10.1186/s13021-025-00369-2.

## Background

 Global warming, driven by excessive greenhouse gas emissions, has become one of the most pressing and critical environmental challenges of the 21 st century. China, as the world’s largest carbon emitter, released over 11.9 billion tons of CO₂ in 2024-approximately 33% of the global total. How to actively promote carbon sequestration and emission reduction to cope with global warming has become an important mission for China to fulfill its international commitments and demonstrate its responsibility as a great power. At the same time, with the improvement of the living standard of the population and changes in the consumption structure, China’s demand for food is growing, and dependence on imports of major agricultural products is becoming more and more serious, so how to effectively guarantee national FS under the constraints of water and soil resources and carbon sequestration and emission reduction (CSER) has become a matter of great importance for the country’s economy and people’s livelihood. However, while guaranteeing a stable supply of food, China has also paid a heavy price, especially for the traditional agricultural model characterized by high inputs, high consumption and high emissions, which has not only exacerbated land degradation and food production instability, but also posed a serious challenge to CSER targets and FS. Agricultural cultivation, as a basic industry that supports the national economy and guarantees national FS, is both an important source of carbon emissions and an important sink for carbon fixation. In recent years, despite China’s active carbon reduction measures, greenhouse gas (GHG) emissions from agricultural activities in China account for about 17% of the country’s total GHG emissions, with carbon emissions from agricultural cultivation accounting for about 50% of the total agricultural carbon emissions, indicating that there is a huge potential for CSER from agricultural cultivation [1, 2]. In 2022, the Government Work Report clearly put forward the requirement of “promoting green development in agriculture”, and in the same year, the Ministry of Agriculture and Rural Development and the National Development and Reform Commission issued the “Implementation Program for CSER in Agriculture and Rural Areas”, which puts forward the objectives, priorities and implementation paths of CSER in agriculture at the national and regional levels. China’s 2024 Government Work Report once again called for “promoting green agricultural development” and further stressed the importance of “strengthening food supply security,” underscoring that ACSER and FS are dual imperatives of modern agricultural practices.

Currently, there is a significant imbalance and disharmony between the ACSER capacity of different food functional areas in China and the guarantee of national FS [3]. For example, the main grain marketing areas have limited CSER capacity of agricultural cultivation due to rapid urbanization, tight arable land resources, and a weak agricultural base, and the supply of food production is mainly dependent on external inputs [4]; Although the main food-producing areas have a strong potential for food production and carbon sinks, the high input, high emission agricultural model is more common, leading to low carbon emission efficiency in agricultural production and serious waste of resources; However, due to the slow increase in the level of agricultural mechanization in food production and marketing areas, a “high-cost, low-efficiency” agricultural model has been formed, which not only restricts the coordinated development of ACSER and FS, but also exacerbates ecological and environmental pressures [5]. Therefore, is there a coupled synergistic relationship between ACSER and FS? How to scientifically measure the level of its coupling synergy and identify its influence mechanism has become one of the current urgent scientific problems. Based on this, exploring the coupling and synergistic relationship between ACSER and FS, and clarifying the internal logic and spatial spillover effects of the coupling and synergistic relationship between the two are of great theoretical and practical significance for promoting the green transformation of agriculture, enhancing the resilience of FS, and formulating region-specific ACSER and FS policies [6]. This study aims to establish a new analytical paradigm and theoretical framework for the coupled synergy between ACSER and FS through systematic measurement, spatial analysis, and mechanism identification. It seeks to provide methodological innovations and practical pathways for the coordinated advancement of agricultural carbon neutrality and carbon peaking goals alongside food security strategies.

## Literature review

In recent years, both domestic and international scholars have made significant progress in the study of the synergistic development between ACSER and FS. However, most existing research primarily focuses on the relationships among agricultural carbon emission intensity, carbon source–sink characteristics, emission reduction pathways, and ecological compensation in relation to FS [7–10]. Relatively few studies have conducted an in-depth exploration of the coupling and coordination relationship between ACSER and FS. In fact, the core content of ACSER and FS is to properly handle the relationship between the two, not only to pay attention to the importance of agricultural cultivation to ensure FS, but also to see the impact of CSER and food production in promoting the realization of the goal of the “dual carbon”; It is important to recognize the paradoxical conflict that exists between ACSER and FS, but also to see the stronger intrinsic synergy that exists between the two, to achieve the unity of the dual-point theory and the focus theory. Currently, ACSER and FS are likely to face conflicts in the short term between increased food production and carbon sequestration reduction, as well as sectoral policy coordination. First, there is a certain contradiction between increased food production and emission reduction. Increased food production often relies on increased agricultural inputs or expansion of arable land, which can easily lead to increased greenhouse gas emissions and weaken CSER. Emission reduction measures, on the other hand, may limit production capacity and result in reduced food production. This contradiction will become more pronounced as soil and water resources are strained. For example, Shirsath and Aggarwal conducted a simulation study on agricultural development in the state of Bihar, India, and systematically analyzed the triple trade-off between “food production, greenhouse gas emission reduction, and financial income”, and the study showed that there is a risk of a decline in agricultural production after the adoption of emission reduction policies [11]. Second, there is also a conflict over CSER in agricultural cultivation. Carbon sequestration in agricultural cultivation refers to the absorption of carbon emissions from agricultural cultivation by crops through photosynthesis and storage in the soil, which in turn increases the soil organic matter content, but soils with a high organic matter content increase CH_4_ emissions from paddy fields and N_2_O emissions from drylands [12]. Finally, there is a mismatch between the policies of the relevant sectors to increase production and reduce emissions. The policies introduced by the authorities responsible for agricultural and rural work are mainly aimed at stabilizing production and supply, increasing income and ensuring FS, which will undoubtedly increase the area of arable land and promote agricultural mechanization through the crowding out of forests, wetlands and grasslands, thus leading to a reduction in carbon sinks and an increase in the consumption of fossil fuels, which will in turn increase carbon emissions. On the other hand, the policies introduced by the ecological and environmental protection sector, which focus on the “dual-carbon” target, are based on prioritizing ecological protection, carbon reduction, and emission reduction. For example, in rice cultivation, the ecological and environmental protection department requires the compression of double-cropped rice planting area and the reduction of straw return to the field, which is conducive to the reduction of CH_4_ emissions, but is not conducive to the high yield of rice. In addition, changes and adjustments in relevant sectoral policies and the degree of profitability of agricultural production entities can also affect the relationship between ACSER and FS, or even conflict.

However, from the point of view of the long-term evolutionary trend of agricultural development, national policy orientation and regional empirical results, the relationship between CSER in agricultural cultivation and FS is more often shown as a strong intrinsic synergistic relationship, which is also one of the important directions of the current sustainable development of agriculture [1]. Firstly, increased CSER in the agricultural cultivation process can help improve the agricultural environment and soil quality, thereby increasing food production and ensuring national FS. For example, Villat et al. systematically reviewed the effects of various agricultural practices such as crop mulching, no-tillage, and organic fertilizer application on soil carbon sequestration, verified the fact that soils positively contribute to the enhancement of carbon sinks, and emphasized that these practices both enhance soil quality and food production [13]. Li et al. revealed the interactive relationship between crop yield increases and soil organic carbon in coastal farmland systems. They found that under climate change, increased grain crop yields positively correlate with enhanced soil carbon storage, indicating that yield enhancement itself may promote organic carbon accumulation, thereby constituting a positive feedback mechanism [14]. Joshi et al. investigated the coupled effects of reduced tillage intensity and yield enhancement. Using long-term soil samples and yield data from multiple states in the central United States, they found that lower tillage intensity helps slow soil carbon saturation while maintaining or increasing yields, thereby promoting organic carbon accumulation [15]. Although the above studies reveal a positive feedback mechanism where increased crop yields promote soil carbon sequestration, they remain constrained by regional limitations, simplified model assumptions, and insufficient management scenarios. These approaches fail to adequately account for spatial heterogeneity and socioeconomic constraints. Kori et al. analyzed the FS situation in South Africa under different agro-environmental conditions through systematic evaluation and other methods, and the study showed that agro-environmental conditions play an important role in ensuring FS [16]. Pang et al. conducted a bibliometric analysis and concluded that agricultural environments and soil characteristics are key factors driving changes in carbon sources and sinks and promoting food production [17]. Secondly, the safeguarding of FS provides support for ACSER. The synergy between the two originates from the emission reduction potential unlocked by agricultural technological innovation and efficient resource utilization. For example, the application of oxygenators and urease inhibitors not only promotes abundant and stable yields of rice and wheat, but also promotes soil carbon sequestration while significantly reducing rice CH_4_ and soil N_2_O emissions through oxidation [18]; Zhang et al. found that CSER in agricultural cultivation under club convergence can effectively promote the positive interaction between CSER and crop yield increase by relying on agricultural technology innovation and optimizing production layout [19]. Rong examined how green technological innovation influences the intensity of agricultural carbon emissions in Jiangsu Province. The findings indicate that such innovation not only directly reduces emission intensity but also generates notable short-term positive spatial spillover effects. In the long run, it plays a protective role in safeguarding food production [20]. Tang et al. found that the scale of agricultural technological innovation not only directly suppresses carbon emissions but also exerts a stronger emission-reducing effect through diversity and structural adjustment mechanisms, accompanied by spatial spillover effects [21]. Consequently, a lag effect and nonlinear relationship may exist between technological innovation and input-output, which should be considered in model design.

Combing through the above literature, it is found that although scholars at home and abroad have conducted extensive research on the measurement of ACSER, FS, the spatiotemporal characteristics and influencing factors, as well as the formulation of related strategies [22–25], a new type of research paradigm that focuses on the synergistic development of the capacity of carbon sequestration and FS of agricultural farming has not yet been formed. And in terms of research scales, most of the existing studies focus on the national or provincial levels. Although research at the national level can reveal overall trends in agriculture, it is difficult to meet the practical needs of localized and disaggregated policymaking; while research at the provincial scale, although it can demonstrate the characteristics of development within a region, tends to sever the spatial correlation between regions and increase the cost of coordinated regional governance. In terms of research methodology, the traditional coupled synergy model suffers from problems such as the aggregation of high values of indicators and the difficulty of revealing actual differences, while it is mostly used for inter-provincial horizontal comparisons and lacks longitudinal consideration of time-series changes. For example, Zheng et al. revealed at the provincial level a coupled evolutionary trend where agricultural carbon emission efficiency and food security progressed from “barely coordinated” to “primitive coordination.” However, the study lacks more regionalized and refined-scale analysis, making it difficult to provide precise guidance for local policy formulation [26]. He et al. explored the coupling mechanisms between agricultural carbon sink efficiency and food security. While offering an innovative perspective, their study primarily employed static coupling models, failing to fully capture temporal variations and spatial spillover effects [27]. Sun et al. examined China as a whole to measure the coupling coordination between food security indices and agricultural carbon emission efficiency, analyzing their trends and influencing factors. They found that the coordination between the two improved over time. The study also revealed that the coupling mechanisms between them exhibit heterogeneity under different government priority scenarios [28]. Although this study incorporates government priority scenarios, its scenario design may be overly macro-level and policy-level in nature, potentially lacking sufficient depth in exploring spatial spillover effects.

**Fig. 1:**
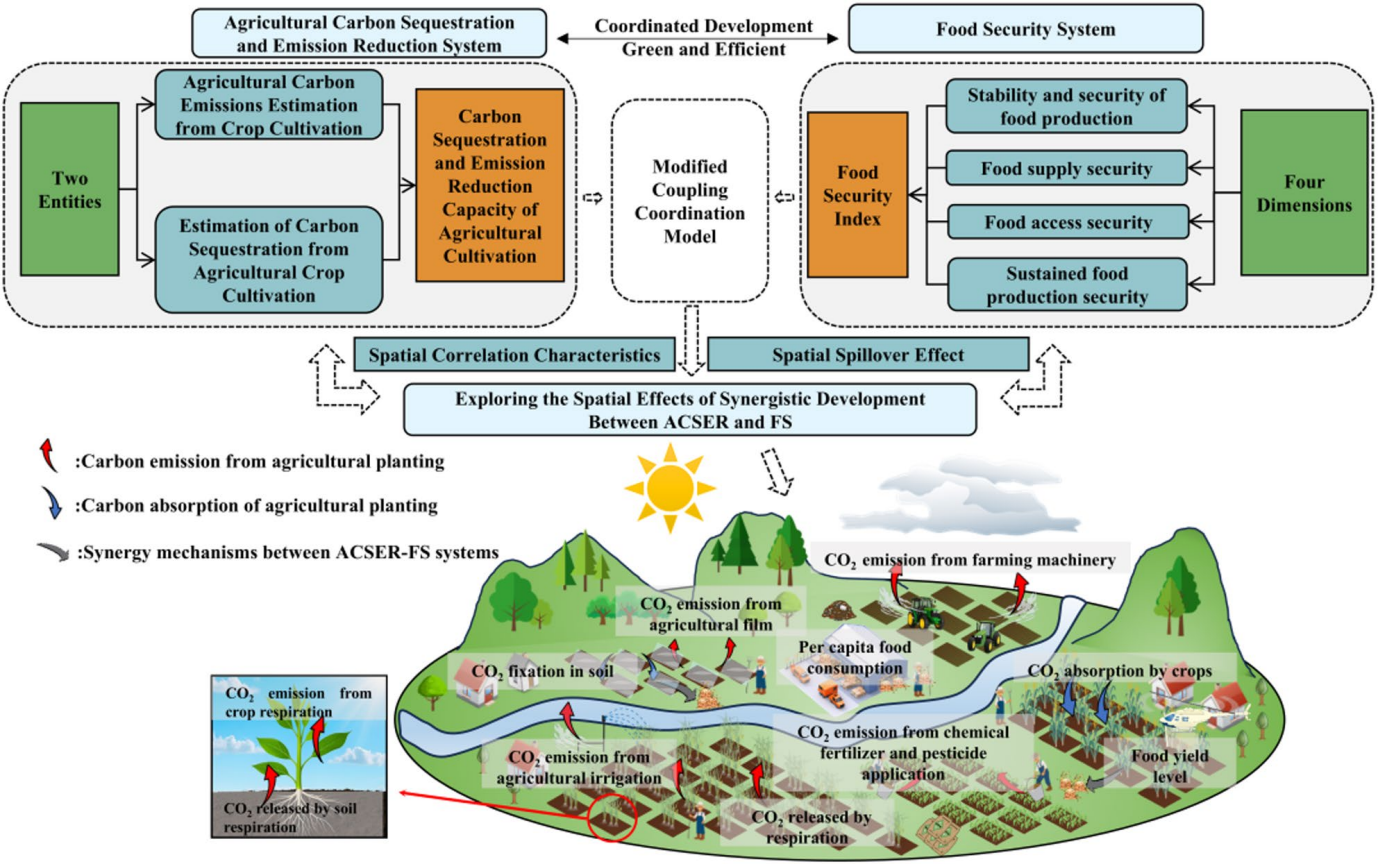
Research framework

The study discusses the relationship between ACSER and FS synergistic development, and its main contributions are: (1) By constructing a framework for analyzing the coupling and synergistic analysis of ACSER and FS, the mechanism of the coordinated development of ACSER and FS is revealed, which enriches the theoretical system of the sustainable development of agriculture; (2) In terms of research scale, by measuring and comparing the development level of synergy between ACSER and FS in different food functional areas in China, data support is provided for the formulation of differentiated CSER and FS policies; (3) In terms of methodology, the introduction of the time-series CCD Modification model significantly enhances the resolution of coupling assessment, strengthens longitudinal dynamic analysis, and expands its utility for policy evaluation. This provides a feasible approach to uncovering the temporal patterns and developmental potential of the synergistic evolution between ACSER and FS. At the same time, the SDM was employed to analyze the influencing mechanisms and spatial spillover effects of the CCD between ACSER and FS in different food functional areas in China. This analysis provides both theoretical and empirical support for understanding the coordinated development of ACSER and FS. It also helps formulate regional linkage strategies and plays an important role in promoting green and low-carbon agricultural development as well as safeguarding food security and sustainability. The specific research framework is shown in Fig. 1.

### Overview of the study area

**Fig. 2:**
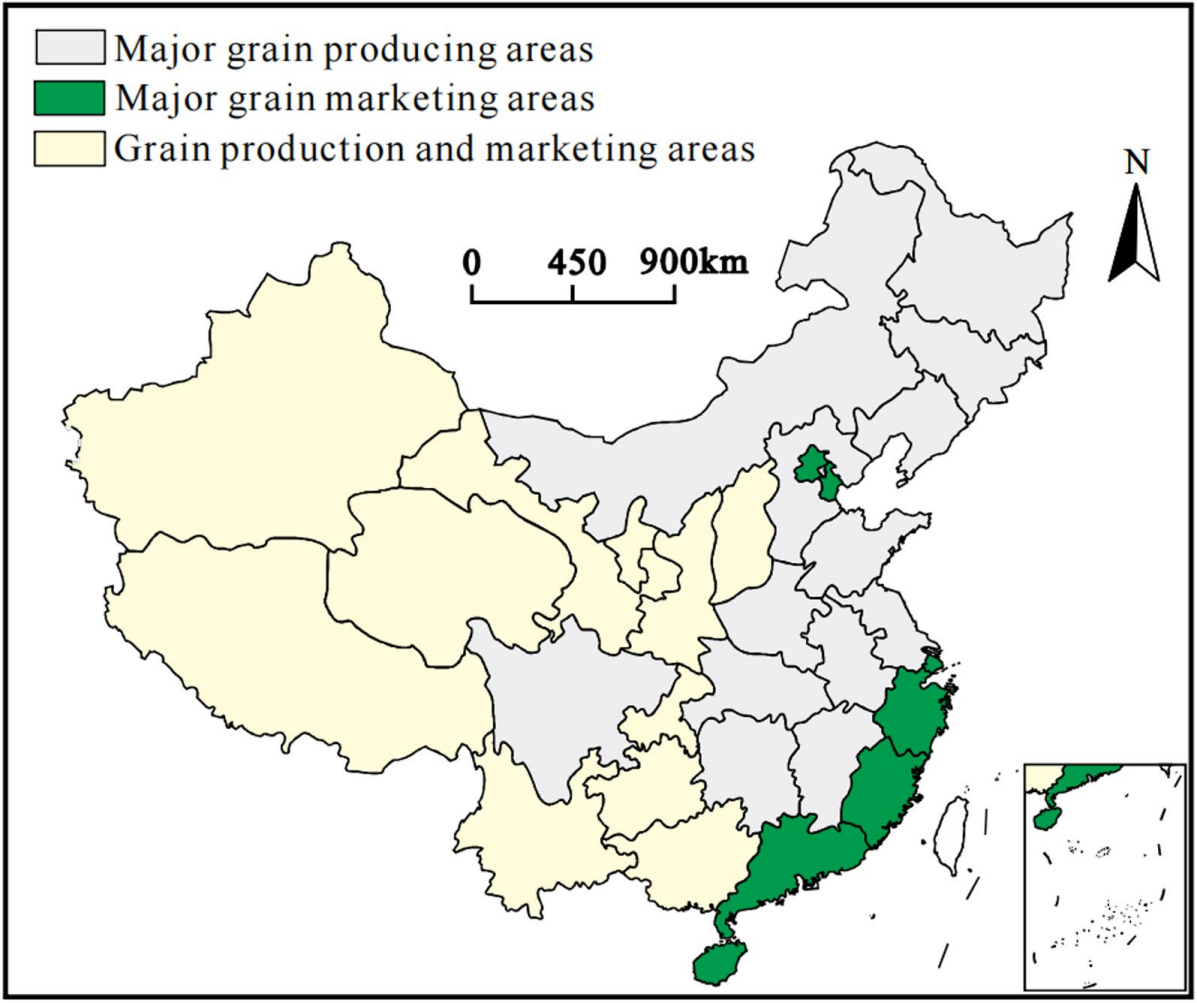
The spatial distribution of China’s three major grain functional areas

In 2017, China’s State Council released the Guiding Opinions on the Establishment of Functional Grain Production Areas and Important Agricultural-Product Production Protection Areas. Drawing on those guidelines-together with each province’s economic development level, farming capacity, resource endowment, and grain supply-and-demand pattern—the country is divided into three functional grain zones (Fig. 2): (1) Main grain production areas, primarily in the central-eastern and northeastern regions, spanning 13 provinces with favorable climates, fertile soils, and abundant water, forming the backbone of national grain supply; (2) Grain production and marketing areas, located in the west and northwest, where harsh terrain and arid climates limit output and production mainly meets local needs; (3) Main grain marketing areas, concentrated along the prosperous southeastern coast, where limited farmland and high population density create a supply gap that must be filled through inter-regional transfers.

## Research methodology

### Estimation of ACSER in crop production

In this study, ACSER is represented by the ratio of carbon absorbed by crops and fixed in soil to the carbon emissions generated from agricultural material inputs and crop growth [22]. The specific calculation formula is as follows:

1 $$\:\text{F}=\frac{{{\text{C}}_{\text{T}}+\text{C}}_{\text{s}\text{o}\text{i}\text{l}}}{{\text{E}}_{{\text{C}\text{O}}_{2}}+{\text{E}}_{{\text{N}}_{2}0}+{\text{E}}_{{\text{C}\text{H}}_{4}}}$$

where $\:\text{F}$ is the CSER index of agricultural cultivation; $\:{C}_{T}$ and $\:{C}_{soil}$ are carbon absorbed by food crops and carbon fixed by agricultural soils, respectively; $\:{E}_{{CO}_{2}}$ is the carbon emissions from agricultural inputs (fertilizers, pesticides, agricultural films, diesel for agricultural machinery, irrigation of agricultural land, ploughing of agricultural land). $\:{E}_{{N}_{2}o}$ refers to the N₂O emissions from crop cultivation, including spring wheat, winter wheat, and maize. $\:{E}_{{CH}_{4}}$ is the emission of CH_4_ from rice cultivation.

### FS estimation methods

The entropy weight extended matter element model is an evaluation method for multi-factor complex problems. By objectively determining weights through entropy weighting and leveraging extensional set theory to quantify complex relationships among indicators, it not only minimizes subjective influences but also offers advantages such as compatibility with diverse data types, enhanced evaluation stability, and improved system adaptability [29]. However, the commonly used Topsis distance function method is highly sensitive to indicator weighting; if weights are allocated unreasonably, it can easily lead to biased results. The Analytic Hierarchy Process (AHP) and the Framework for Participatory Impact Assessment (FoPIA) models are susceptible to participants’ subjective factors, requiring professional guidance and coordination. The Data Envelopment Analysis (DEA) model has certain limitations in handling negative outputs and selecting indicators [23]. In view of the complexity of the FS evaluation itself and the incoherence of the indicators, this paper utilizes the entropy weight extended matter element model to measure the level of FS, and the specific steps refer to the literature [30, 31]. In addition, to systematically portray the synergistic relationship between ACSER and FS, this study constructed a comprehensive evaluation index system of ACSER and FS (ACSER-FS) on the basis of the previous research (Table 1). In terms of the selection of factors influencing the CCD of ACSER and FS, this study combines the key driving mechanisms for the development of ACSER and FS systems [22, 23], and selects seven core variables as explanatory variables: Proportion of arable land area (L), total power of agricultural machinery (M), pesticide application (P), fertilizer application (F), CO₂ uptake by major Crops (CA), grain yield level (Y), and per capita food possession (FP).

**Table 1:** ACSER-FS evaluation indicator system

System	Subsystem	Evaluation indicators	Attribute
ACSER	Agricultural Carbon Emissions Estimation from Crop Cultivation	Carbon emissions from agricultural inputs	Negative
		N_2_O emissions from crops (spring wheat, winter wheat and maize)	Negative
		Rice CH_4_ emissions	Negative
		Carbon emissions from soil respiration	Negative
	Estimation of Carbon Sequestration from Agricultural Crop Cultivation	Carbon uptake by food crops	Attribute
		Carbon sequestration in agricultural soils	Attribute
FS	Food supply security	Grain yield level: total grain production/provincial area	Attribute
		Cultivated land area ratio: Cultivated land area/province area	Attribute
		Total power of agricultural machinery: power of agricultural machinery/cultivated land area	Attribute
	Food access security	Per capita food possession: total food production/resident population in the province	Attribute
	Stability and security of food production	Coefficient of food damage volatility: area affected/area sown with crops	Negative
		Food production volatility coefficient:$\:({Y}_{t}-{Y}_{t}^{{\prime\:}})/{Y}_{t}^{{\prime\:}}$	Negative
	Sustained food production security	Amount of agricultural film applied: Amount of agricultural film applied/area sown with crops	Negative
		Pesticide application: pesticide application/area sown to crops	Negative
		Diesel application: diesel application/area sown with crops	Negative
		Fertilizer application: Fertilizer application/crop sown area	Negative

$\:{\text{Y}}_{\text{t}}$ in the coefficient of food production volatility denotes total food production in year t, and $\:{\text{Y}}_{\text{t}}^{{\prime\:}}$ denotes the five-year moving average of food production

### Modified coupling coordination degree modeling

While traditional coupling coordination models can characterize interactions between systems, they exhibit limitations in practical applications—such as the clustering of high-value indicators and insufficient revelation of temporal differences-making it difficult to accurately reflect the dynamic synergistic evolution of agricultural carbon sequestration and food security systems. Given this, this paper draws upon the research findings of Wang et al. [32], introducing time-series correction coefficients to construct an enhanced coupling coordination model. This approach offers significant advantages in improving coupling resolution, strengthening longitudinal dynamic analysis, and expanding policy evaluation capabilities. It aims to characterize the dynamic temporal evolution of the agricultural carbon sequestration and food security system. While maintaining structural stability, the model dynamically adjusts the relative magnitude of changes between systems across different periods, thereby enhancing its discriminatory power and sensitivity in longitudinal comparisons. Compared to other measurement methods such as grey relational analysis, this model not only dynamically characterizes the co-evolution patterns between the two systems but also exhibits higher discrimination and explanatory power. It provides a more scientific and feasible analytical tool for revealing the temporal patterns and potential pathways of synergistic development between ACSER and food security [33].

In terms of temporal variation, the calculation formula for the system development adjustment coefficient is as follows:

2 $$\begin{aligned}&\:{r}_{it}^{\left(\alpha\:\right)}=\frac{\overline{{X}_{it}^{\left(\alpha\:\right)}}-\overline{{X}_{i(t-1)}^{\left(\alpha\:\right)}}}{\overline{{X}_{i(t-1)}^{\left(\alpha\:\right)}}},\:{R}_{\left(\alpha\:\right)}\\&=\frac{\sum\:_{t={t}_{min}}^{{t}_{max}}\sum\:_{i=1}^{m}{W}_{i}^{\left(\alpha\:\right)}\times\:{r}_{it}^{\left(\alpha\:\right)}}{{t}_{max}-{t}_{min}}\end{aligned}$$

where $\:t$ is the year and takes the value 2000–2023; $\:\alpha\:$ represents the system, when $\:\alpha\:=1$, $\:\alpha\:$ represents the agricultural cultivation CSER system, and when $\:\alpha\:=2$ is the FS system;$\:\stackrel{-}{{X}_{it}^{\left(\alpha\:\right)}}$ denotes the mean value of the data of each indicator in the system; $\:{r}_{it}^{\left(\alpha\:\right)}$ is the growth rate of the system’s data for each indicator in year $\:t$ compared to the previous year; $\:{W}_{i}^{\left(\alpha\:\right)}$ is the weight of each indicator in the system; $\:{R}_{\left(\alpha\:\right)}$ is the time-varying system development adjustment coefficient. When $\:{R}_{\left(\alpha\:\right)}$ is large, it indicates rapid system evolution, and the modified coupling coordination degree becomes more sensitive to changes during this phase. When $\:{R}_{\left(\alpha\:\right)}$ is small, it indicates that the system is approaching stability, with reduced fluctuations in the adjusted coupling coordination degree. This avoids the issue of “high values clustering and low values being masked” found in traditional models.

The formula for calculating the adjusted system development level is as follows:

3 $$\:{U}_{jt}^{{\prime\:}\left(\alpha\:\right)}={U}_{jt}^{\left(\alpha\:\right)}\times\:{(1-{R}_{\left(\alpha\:\right)})}^{{t}_{max}-t}$$

where $\:{U}_{jt}^{\left(\alpha\:\right)}\:$denotes the pre-adjustment development level of the system in year $\:t$ in region $\:j$. The calculation of the pre-adjustment development level of the agricultural cultivation CSER system is derived from Eq. (1), and the calculation of the development level before the FS system adjustment can be found in the FS estimation section; $\:{U}_{jt}^{{\prime\:}\left(\alpha\:\right)}$ is then the adjusted system development level; $\:{R}_{\left(\alpha\:\right)}$ has the same meaning as in Eq. (2).

Based on the adjusted level of system development, the modified CCD formula is:

4 $$\begin{aligned}&\:{C}_{jt}=\sqrt{\left[1-\sqrt{{({U}_{jt}^{{\prime\:}\left(2\right)}-{U}_{jt}^{{\prime\:}\left(1\right)})}^{2}}\right]\times\:\frac{{U}_{jt}^{{\prime\:}\left(2\right)}}{{U}_{jt}^{{\prime\:}\left(1\right)}}}\\&=\sqrt{\left[1-({U}_{jt}^{{\prime\:}\left(2\right)}-{U}_{jt}^{{\prime\:}\left(1\right)})\right]\times\:\frac{{U}_{jt}^{{\prime\:}\left(2\right)}}{{U}_{jt}^{{\prime\:}\left(1\right)}}}\end{aligned}$$

5 $$\:{T}_{jt}=\beta\:{U}_{jt}^{{\prime\:}\left(1\right)}+\gamma\:{U}_{jt}^{{\prime\:}\left(2\right)},{D}_{jt}=\sqrt{{C}_{jt}+{T}_{jt}}$$

$\:{D}_{jt}$ is the coupled coordination degree of ACSER and FS in year $\:t$ in region $\:j$; $\:{T}_{jt}$ is the composite harmonization index for region $\:j$ in year $\:t$; $\:\alpha\:$ and $\:\beta\:$ are coefficients to be determined, and since ACSER and FS are equally important in the synergistic development process, $\:\alpha\:$ and $\:\beta\:$ are taken as 0.5; $\:{C}_{jt}$ represents the system coupling in year $\:t$ in region $\:j$, C ∈ [0,1]. The method of CCD class division is based on the CCD class division method of Fan et al. [34], according to the 10 copies of the CCD of equal probability differentiation, and finally gets the value of the CCD interval of each class as Table 2.

**Table 2:** ACSER and FS coupling harmonization level classification

Level	Range of CCD	Stage	Stage classification	Level	Range of CCD	Stage	Stage classification
1	[0.000, 0.419)	Dysfunction	Extreme Dysfunction	6	[0.550, 0.573)	Coordination	Awkward coordination
2	[0.419, 0.462)		Severe Dysfunction	7	[0.573, 0.596)		Elementary coordination
3	[0.462, 0.499)		Moderate Dysfunction	8	[0.596, 0.627)		Intermediate coordination
4	[0.499, 0.527)		Mild Dysfunction	9	[0.627, 0.675)		Good coordination
5	[0.527, 0.550)		Marginal Dysfunction	10	[0.675, 1.000]		Quality coordination

#### Spatial correlation analysis

In order to explore whether there are overall spatial correlations and local aggregation characteristics of the coupled synergy of ACSER and FS, this paper selects the global Moran index ($\:{I}_{G}$) and the local Moran index ($\:{I}_{i}$) to measure them, and the calculation formulas are as follows, respectively:

6 $$\:{I}_{G}=\frac{n\sum_{i=1}^{n}\sum_{j=1}^{n}{W}_{ij}\left({x}_{i}-\stackrel{-}{x}\right)\left({x}_{j}-\stackrel{-}{x}\right)}{\sum_{i=1}^{n}\sum_{j=1}^{n}{W}_{ij}\sum_{i=1}^{n}{\left({x}_{i}-\stackrel{-}{x}\right)}^{2}}$$

7 $$\:{I}_{i}=\frac{n\left({x}_{i}-\stackrel{-}{x}\right)}{\sum{\left({x}_{i}-\stackrel{-}{x}\right)}^{2}}\sum_{i=1}^{n}{w}_{ij}{(x}_{j}-\stackrel{-}{x})$$

where $\:{x}_{i}$ and $\:{x}_{j}$ are the CCD of ACSER and FS for spatial units $\:i$ and $\:j$, respectively; $\:\stackrel{-}{x}$ is the average value of CCD for the whole study area; $\:{w}_{ij}$ represents the economic-geographic weighting matrix, as it simultaneously reflects both geographic proximity and the strength of economic ties between regions. This matrix accounts for spatial similarities in natural resources and climatic conditions while also capturing the economic interconnections in agricultural inputs, outputs, and technology diffusion; $\:n$ is the number of spatial cells.

## Spatial durbin model

### Model building

Since the Spatial Durbin Model (SDM) can comprehensively reveal the spatial lag effect of the dependent variable and the spatial spillover effect of the explanatory variables, the SDM was selected to analyze the influencing factors of the coupled synergy of ACSER and FS in this paper. The root reference Elhorst [35], assumes that the expression for SDM is:

8 $$\begin{aligned}\:{ln}{D}_{jt}&={\rho\:Wln}{D}_{jt}+\sum_{k=1}^{7}{\beta}_{k}{ln}{X}_{kjt}\\&+\sum_{k=1}^{7}{\theta}_{k}Wln{X}_{kjt}+{\mu}_{j}+{\lambda}_{t}+{\epsilon}_{jt}\end{aligned}$$

where $\:{D}_{jt}$ denotes the CCD of ACSER and FS coupling in year $\:t$ in region $\:j$; $\:\rho\:$ denotes the spatial autoregressive coefficient; $\:W$ is the spatial weight matrix, same as $\:{w}_{ij}$ in Eq. (6); $\:{\beta\:}_{k}$ is the local regression coefficient of the explanatory variables; $\:{X}_{kjt}$ denotes each explanatory variable ($\:L$, $\:M$, $\:P$, etc.) for year $\:t$ in region $\:j$; and $\:{\theta\:}_{k}$ is the coefficient of the spatial lag term of the explanatory variables; $\:{\mu\:}_{j}$, $\:{\lambda\:}_{t}$, and $\:{\epsilon\:}_{jt}$ denote region fixed effects, time effects, and random error terms, respectively, and all variables are expressed in logarithmic form in order to eliminate the effects of heteroskedasticity.

### Estimation of direct and indirect effects

In the SDM model, the explanatory variables not only have an impact on local outcomes but also act on neighboring areas through spatial lag terms. In this paper, we refer to the effect decomposition method based on the partial derivative matrix proposed by Lesage and Pace [36] and Elhorst to divide the total effect into direct effect and spatial spillover effect, which is calculated as follows:

 

9 $$\begin{aligned}&\text{Direct effect:}={DE}_{ln{X}_{k}}\\&=\frac{1}{n}\varvec{t}\varvec{r}\left[{\left({D}_{jt}-\rho\:W\right)}^{-1}\left({\beta\:}_{k}{D}_{jt}+{\theta\:}_{k}W\right)\right]\end{aligned}$$

 

10 $$\begin{aligned}&\text{Indirect effects:}=\:{IE}_{ln{X}_{k}}\\&=\frac{1}{n}{1}^{{\prime\:}}\left[{\left({D}_{jt}-\rho\:W\right)}^{-1}\left({\beta\:}_{k}{D}_{jt}+{\theta\:}_{k}W\right)\right]1-{DE}_{ln{X}_{k}}\end{aligned}$$

where **tr()** denotes the trace of the matrix, i.e., the sum of the main diagonal elements, which is used to measure the average effect of the independent variable on the dependent variable and represents the theoretical basis of the direct effect; Eq. (10) where **1** is the all-1 vector and **1′A1** denotes the sum of all elements of matrix A. Summing the spatial effects across all regions is achieved by multiplying left by **1′** and right by **1**, and averaged to give the total effect value of the variable in the spatial dimension.

## Data Source

This study employs provinces in mainland China as its basic analytical units. The data concerning food security and carbon sequestration through agricultural cropping primarily originate from the China Statistical Yearbook, China Rural Statistical Yearbook, and provincial (autonomous region, municipality) statistical yearbooks and annual bulletins spanning 2001–2024. The indicators employed include cultivated land area, provincial territory area, disaster-affected area, total power of agricultural machinery, fertilizer usage, pesticide usage, agricultural plastic film usage, diesel consumption, effectively irrigated area, grain crop planting area, and grain production [37–40].

Considering data integrity and availability, the study excludes Hong Kong, Macau, and Taiwan regions. For minor data gaps in specific provinces—such as missing agricultural film and diesel consumption data for Xinjiang and Hainan in 2001 and 2005, and missing agricultural film data for Gansu in 2002 and 2006—this paper employs linear smoothing interpolation to fill the gaps. This method assumes a linear trend between the missing value and the data from adjacent years. By fitting the slope of the change between the preceding and subsequent periods, it estimates the intermediate value to ensure data continuity and consistency within the time series.

## Results

### Spatial-temporal characteristics of the coupled synergies of ACSER and FS

**Fig. 3:**
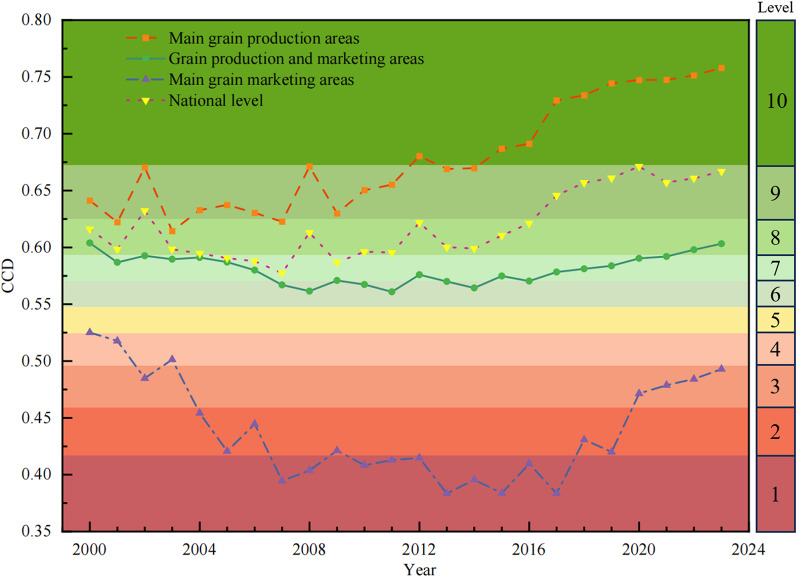
Changes in coupled synergy levels of ACSER and FS in different food functional areas in China; The classification levels in the figure refer to Table 2

Figure 3 shows that, at the national level, the coupling of ACSER and FS fluctuates from 2000 to 2023, showing a gradual evolution from “intermediate coordination” to “good coordination”. From 2000 to 2004 (except 2002) and again from 2010 to 2015, the level of “intermediate coordination” prevailed—indicating that during these periods the synergistic development of ACSER and FS was still in its infancy, and the degree of synergy had not yet become significant. In 2002 and 2016–2023, the coupling synergy level was raised to “good coordination”, indicating that the coordinated development of ACSER and FS has entered a stage of optimization and improvement, with the degree of coupling synergy increasing and the synergistic benefits gradually being released. However, the phenomenon of “elementary coordination” appeared in 2005–2009, which may be due to the influence of policy, technology, or resource mismatch and other factors in this period [41]. The coupling efficiency of ACSER and FS is relatively low, resulting in the existence of a certain unevenness and instability in the operation of ACSER and FS. Overall, the level of synergy between ACSER and FS in China’s agriculture has been rising steadily, showing a path of “elementary-intermediate-good”, indicating that the green transformation of agriculture and FS has gradually formed a synergistic momentum under the background of the “dual-carbon” approach.

With regard to different food functional areas, the synergy between ACSER and FS in the main producing areas will continue to improve during the period from 2000 to 2023, showing a development trend of “localized oscillation and stable improvement”. However, from 2000 to 2005, the coupling synergy between ACSER and FS in the main food-producing areas fluctuated considerably, and although the overall situation was above the level of “elementary coordination”, the synergy between the two was relatively weak due to the fact that the green agricultural technology had not yet been popularized, the institutional mechanism was not sound, and the knowledge of the concept of carbon sequestration was insufficient, which resulted in certain stage fluctuations. With the continuous strengthening of national policies on green agriculture and FS, especially the implementation of strategic measures such as “hiding food in the land and food in technology”, the level of synergy in the main producing areas improved significantly from 2007 to 2012, and agricultural production was gradually transformed to eco-efficiency, with the coupling mechanism becoming more mature [42]. Since the “14th Five-Year Plan”, the main producing areas have continued to make efforts in green production, energy saving, and emission reduction, and production capacity guarantee, further optimizing the synergistic relationship and significantly improving the CCD, which is higher than 0.750, generally reaching the level of “quality coordination”. This shows that the main production area, as the core area of China’s FS, has basically built up a development model that emphasizes both eco-friendly agriculture and FS, and the endogenous synergistic capacity of the agricultural system for CSER and FS has been continuously strengthened, which provides a typical model for the coordinated development of national FS and low-carbon agriculture under the goal of “dual-carbon”. In the production and marketing areas, the overall synergy of ACSER and FS coupling from 2000 to 2023 is “moderately low and steadily increasing”. As can be seen in Fig. 3, the overall level of synergy in the region was low at the beginning, and the CCD has been at the level of “awkward coordinated” or below for a long period of time, with strong fluctuations, reflecting the fact that the linkage between ACSER and FS is not yet sound, and the synergistic effect is relatively limited. Since 2010, the coupling level of production and marketing areas has gradually stabilized and entered a continuous optimization channel during the “13th Five-Year Plan” period, with the coupling level moving into the “elementary coordination” level range, and the foundation of synergy has been consolidated. In recent years, the level of coordination between ACSER and FS in the production and marketing areas is still lower than that in the main production areas and the national average, but the overall trend has been positive, indicating that the degree of match between green agricultural development and FS in the region has gradually increased, and coupling relationships tend to be synergistic. Overall, although the production and marketing areas have a low starting point in the synergistic evolution of ACSER and FS, they have the potential to improve steadily, and in the future, they need to continue to optimize the structural conditions and policy appropriateness in order to consolidate their synergistic development foundation.

In the main marketing area, the overall CCD of ACSER and FS from 2000 to 2023 is at a relatively low level, hovering in the “mild dysfunction” category or below for a long period of time, which reflects the relatively weak foundation for the synergistic development of ACSER and FS. Figure 3 shows that the region as a whole was in a state of “mild dysfunction” to “moderate dysfunction” at the beginning of the study, with large fluctuations in the degree of coupling, and even close to the threshold of “severe dysfunction” in some years. This indicates that the ability of CSER from agricultural cultivation to support FS is limited, and the coordination mechanism within the region is not yet sound. Nevertheless, the level of synergy in the main marketing areas has risen since 2016, with the CCD reaching 0.410, and will enter “moderate dislocation” and continue to stabilize after 2020, reflecting the initial results achieved in some areas in the green transformation of agriculture and the construction of food control capacity. Overall, due to weak agricultural foundations, limited arable land resources, and a high degree of external dependence, the main sales regions exhibit insufficient linkage and low synergy efficiency between ACSER and FS. However, under the guidance of green policies and the promotion of urban ecological agriculture pilot projects, the CCD shows a rising trend. In the future, the main marketing areas should strengthen the linkage between the agricultural and distribution systems, improve the efficiency of resource allocation, and optimize the FS mechanism in order to achieve a higher level of synergistic development of ACSER and FS.

**Fig. 4:**
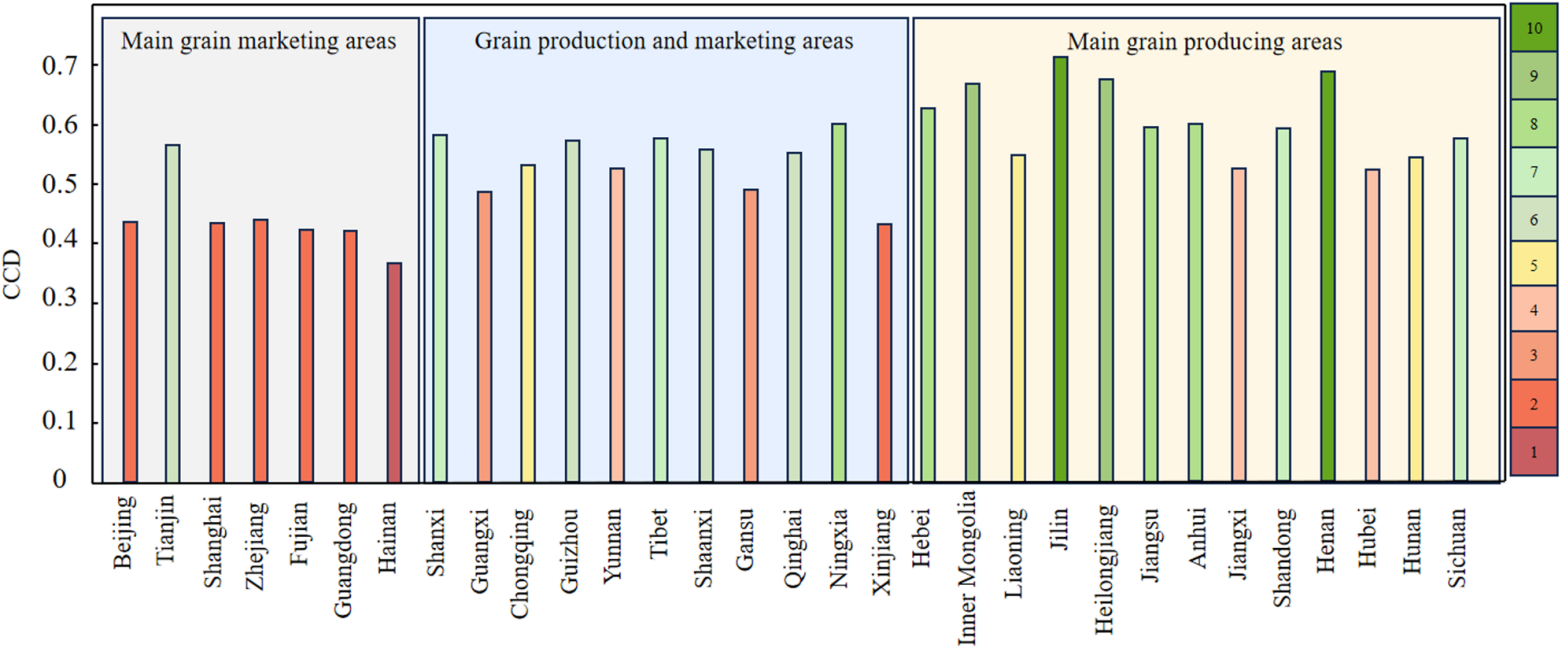
Average CCD of ACSER and FS across Different Food Functional Areas in China (2000–2023); The classification levels in the figure refer to Table 2

Figure 4 shows that the CCD of ACSER and FS in the three major food functional regions from 2000 to 2023 is significantly different. The main production regions demonstrate a relatively high overall level of coordination. For instance, the CCD values between ACSER and FS in provinces such as Heilongjiang, Shandong, and Henan are 0.673, 0.592, and 0.687, respectively, consistently falling within the ‘well-coordinated’ or higher category. This is attributed to their strong agricultural foundations, abundant arable land resources, and effective implementation of green agricultural policies, which have jointly promoted improvements in both CSER and FS. The production and marketing areas show significant regional differentiation, such as Shaanxi and Gansu, etc., which gradually improve the level of synergy through green agricultural inputs and ecological restoration, but Xinjiang and Guangxi, etc., are limited by fragile natural conditions, a weak agricultural base and ecological constraints, with CCD values of 0.432 and 0.489, respectively, which are at a lower level; The main sales regions generally exhibit low coupling levels, with most CCD values below 0.450. Megacities such as Beijing and Shanghai, constrained by limited agricultural land and insufficient ecological support capacity, typically fall into a state of ‘discoordination’. In contrast, Tianjin has significantly improved its coordination level in recent years, benefiting from increased investment in agricultural technology and institutional innovation, gradually approached the ‘well-coordinated’ category. Overall, the coupling and synergistic patterns of different functional areas are constrained by resource endowments, agricultural input structures, and policy orientations, and differentiated development strategies should be formulated according to local conditions to promote the coordinated integration of green transformation of agriculture and FS guarantee.

### Characteristics of the spatial correlation of coupled synergies of ACSER and FS

To further explore the spatial correlation characteristics of the coupled synergy of ACSER and FS, this paper measured the global Moran’s I index for 2000, 2007, 2015, and 2023. At the national level, the global Moran’s I index increased from 0.086 in 2000 to 0.337 in 2023 and passed the significance test (*P* < 0.05) after 2007, indicating a significant positive spatial correlation between ACSER and FS coupling in the whole country, which may be related to the policy convergence at the national level, synergy effect generated by the promotion of the green agriculture and the optimization of the layout of food functional areas. From a regional perspective, the temporal evolution of the global Moran’s I index in both major grain-producing and production-marketing balance areas exhibits a similar pattern. Specifically, the index shifts from negative to positive over time and continues to rise, suggesting a narrowing spatial disparity in the coordinated development of agricultural cultivation, carbon sequestration, emission reduction, and FS. This also reflects an increasing positive spatial autocorrelation across these functional zones. However, most of them failed the significance test, which might be related to the uneven differences in agricultural development and the significant variations in resource endowments within the functional areas. The global Moran’s I index for the main grain sales regions exhibits a consistent downward trend, decreasing from 0.576 in 2000 to −0.206 in 2023, indicating an increasing tendency toward negative spatial correlation. Furthermore, none of the four time points reach statistical significance, suggesting a pronounced spatial heterogeneity in the coupling coordination between agricultural carbon sequestration and FS in these regions. This may be largely attributed to factors such as accelerated urbanization and the non-agricultural transformation of land, which have significantly weakened regional coordination levels (Table 3).

**Table 3:** Global moran’s I of CCD between ACSER and FS in the three major food functional areas of China

Year	National level			Main grain production areas			Grain production and marketing areas			Main grain marketing areas		
	I_G_	Z	*P*-value	I_G_	Z	*P*-value	I_G_	Z	*P*-value	I_G_	Z	*P*-value
2000	0.086	1.080	0.140	−0.080	−0.180	0.493	−0.295	−0.909	0.182	0.576	1.421	0.078
2007	0.156	1.688	0.046	0.042	0.674	0.250	−0.376	−1.195	0.116	0.483	1.423	0.077
2015	0.312	3.088	0.001	0.225	1.622	0.052	0.127	1.006	0.157	−0.303	−0.338	0.368
2023	0.337	3.295	0.000	0.392	2.525	0.006	0.192	1.325	0.093	−0.206	−0.119	0.453

**Fig. 5:**
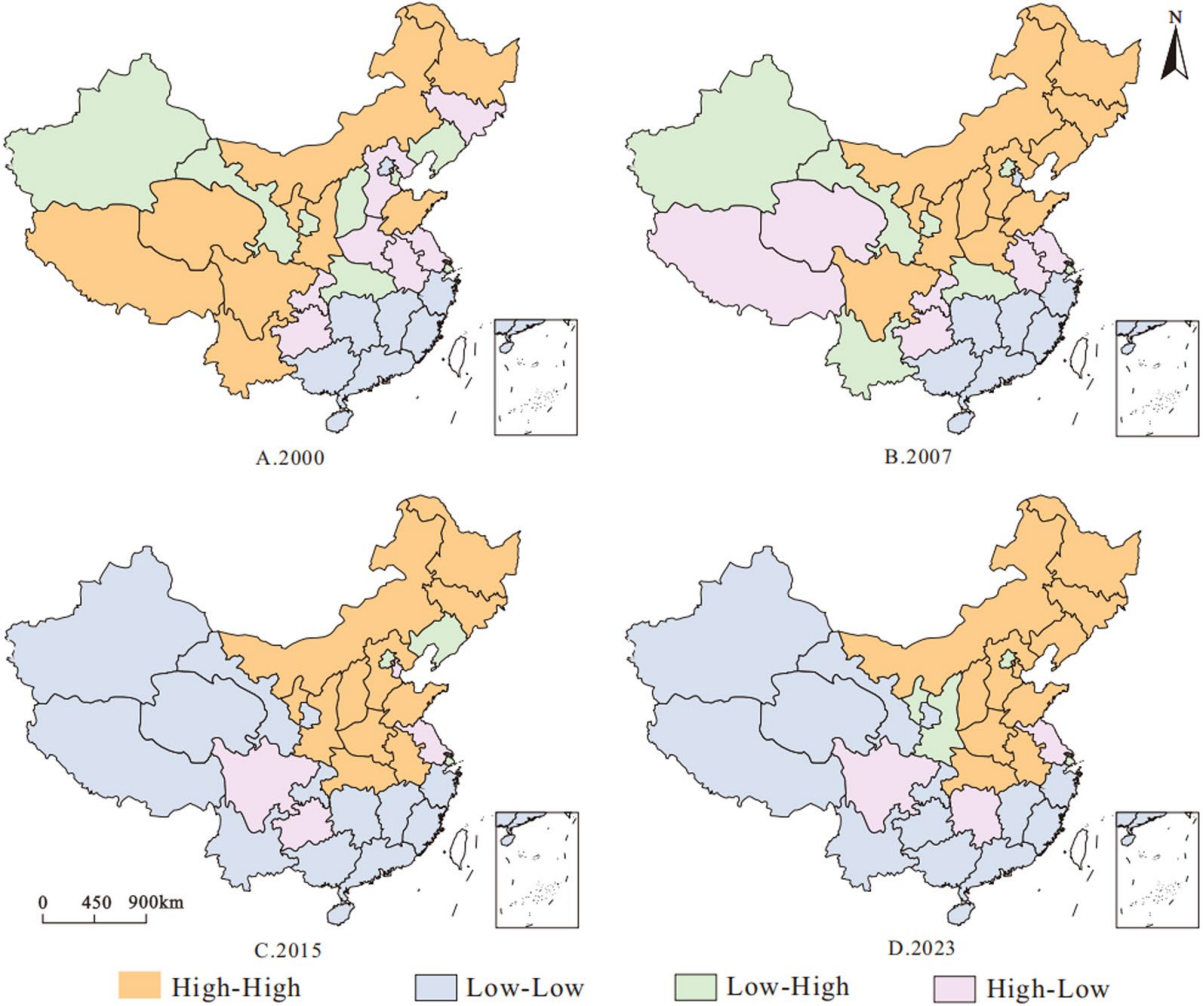
LISA Cluster Distribution of the CCD between ACSER and FS in China; High–High: high values with high neighbors; Low–Low: low values with low neighbors; High–Low: high values surrounded by low; Low–High: low values surrounded by high

Additionally, the spatial distribution of carbon sequestration and emission reduction, coupled with food security in China’s agricultural cultivation, exhibits distinct spatial clustering patterns (Fig. 5). All results passed statistical tests at the 5% significance level (*p* < 0.05), maintaining an overall “higher in the north, lower in the south” pattern. However, the spatial clustering types across different years show phased evolutionary characteristics. In 2000, ‘high-high‘ agglomerations at the national level were mainly concentrated in the northern regions, including the main grain-producing areas of Inner Mongolia, Heilongjiang, and Shandong, indicating a high level of synergy between ACSER and FS in these regions. Meanwhile, southern provinces such as Fujian, Jiangxi, Hunan, and Guangdong are identified as ‘low–low’ clustering areas, reflecting a low level of coordination between ACSER and FS, and indicating the emerging issue of regional development imbalance in southern China. By 2007, the ‘high-high’ agglomeration area had further expanded from northern China to the central-eastern region, with Shaanxi, Hebei, Liaoning, Jilin and other regions showing significant increases in synergism, while high-value areas such as Tibet, Yunnan and other high value areas appeared to be of the ‘high-low’ or ‘low-high’ type, indicating that there was a risk of local coupling imbalance at that stage, which tended to be heterogeneous. At the same time, some of the low-synergy areas in the South have slightly converged, and the spatial clustering effect has increased. By 2015, the spatial agglomeration pattern had undergone a significant transformation. The original “high–high” agglomeration areas had markedly diminished, and apart from Henan and Shandong, which continued to exhibit strong synergy, the level of coordination in the northwestern and northeastern regions had declined, with some areas shifting toward the “low–low” type. In 2023, the spatial pattern stabilizes, but regional differences further intensify. The ‘high-high’ agglomeration area regrouped in the Beijing-Tianjin-Hebei and the Yellow-Huaihai Plain, and the synergistic effect of Shandong, Henan, Anhui, and other places once again came to the fore, forming a stable and dominant region. On the contrary, Guizhou and Hunan have evolved into ‘low-low’ or ‘high-low’ types, showing a tendency to disconnect the development of FS and CSER, and need to pay attention to the construction of synergistic mechanisms and strategies to make up for the shortcomings. Overall, the spatial evolution of the synergy between ACSER and FS shows a pattern of ‘stable high coordination in the north and fluctuating low-to-moderate coordination in the south’. From 2000 to 2023, the concentration trend of high-value agglomerations increases, while low-value regions show some diffusion, implying that future policy formulation should focus on the integration and optimization of regional synergies in order to promote balanced and efficient development of the system.

### Spatial spillovers of coupled synergies between ACSER and FS

#### Selection and testing of spatial measurement models

The aforementioned findings indicate that there are significant overall spatial autocorrelation and local aggregation characteristics in the coupled synergy of ACSER and FS, so exploring the spatial effects of the coupled synergy of ACSER and FS is needed. Drawing on the idea of Elhorst [35], the spatial Lagrangian (LM) test is first utilized to determine whether the coupled synergy of the two has spatial error effects and spatial lag effects; Then, the Hausman test is conducted to determine whether the spatial regression model should adopt fixed effects or random effects. Subsequently, the likelihood ratio (LR) test and Wald test are employed to further assess whether the SDM degenerates into a Spatial Error Model or a Spatial Lag Model. Finally, the appropriate spatial measurement model for this study is determined based on the test results (Table 4).

**Table 4:** LM test, Hausman test, Wald test, and LR test results

Test Method	Test Statistic	*p*-value
*LM test no spatial error*	611.967***	0.000
*Robust LM test with no spatial error*	532.075***	0.000
*LM test no spatial lag*	81.421***	0.000
*Robust LM test with no spatial lag*	1.530***	0.000
*Hausman*	10.68	0.220
*LR Lag*	212.66***	0.000
*LR Err*	172.56***	0.000
*Wald Lag*	38.40***	0.000
*Wald Err*	44.58***	0.000

***, **, and * denote significance at the 1%, 5%, and 10% levels, respectively

The LM test and Robust-LM test showed that the coupled synergy of ACSER and FS passed the significance test at the 1% level, indicating that the choice of the SDM was justified; However, the Hausman test is not significant, i.e., rejecting the hypothesis that ‘there is no systematic difference between fixed-effects and random-effects models’, and finally, combining the LR test and the Wald test, this paper adopts the SDM with random effects for analysis.

### Estimation and analysis of SDM for CCD of different food functional areas in China

**Table 5:** National-Level SDM Estimation results of CCD

SDM	lnL	lnM	lnP	lnF	lnCA	lnY	lnFP
Analysis Results	−0.112***	0.022***	−0.056***	−0.074***	0.295***	−0.062**	0.024
	(0.028)	(0.006)	(0.007)	(0.012)	(0.022)	(−0.028)	(−0.018)
*ρ*	0.442***(0.039)						
*σ* ^*2*^	0.001***(0.000)						
Sample Size	744						
R^2^	0.335						

***, **, and * denote significance at the 1%, 5%, and 10% levels, respectively. Values in parentheses are t-statistics

As shown in Table 5, an empirical analysis of the coupling coordination relationship between ACSER and FS in China is conducted using the SDM. The resulting coefficient of determination (R²) is 0.335, indicating that the model has a good overall fit and demonstrates strong explanatory power. The coefficient of the spatial lag term is 0.442 and passes the 1% significance test, indicating that its CCD has a significant spatial spillover effect, which is consistent with the research theory of LeSage and Pace, and also fits the reality background of China’s strong regional agricultural policy linkage [36]. From the results of variable regression, the regression coefficient of CO₂ uptake by major crops (lnCA) was 0.295, which had the strongest positive effect among all variables and reached a highly significant level, indicating that improving the efficiency of crop CO_2_ uptake in the atmosphere not only helps to enhance the production capacity of crops, but also effectively improves the level of carbon sinks in agricultural cultivation, which is an important path to realize the synergistic promotion of green transformation of agriculture and guarantee FS, which further confirms the positive role of policy measures such as ‘precise fertilization’, ‘efficiency enhancement and emission reduction’, etc. in promoting the development of green agriculture [43]. The total power of agricultural machinery (lnM) also shows a significant positive effect (coefficient of 0.022), indicating that the increase in the level of agricultural mechanization not only improves production efficiency but also reduces the waste of resources in traditional high-carbon agriculture, thus promoting a synergistic win-win situation between the low-carbon transition and the improvement of food production. On the contrary, the regression coefficients of pesticide application (lnP) and fertilizer application (lnF) were − 0.056 and − 0.074, respectively, which were both significantly negative at 1% level, indicating that the traditional high inputs not only failed to effectively promote the coupling synergy of the two in the current agricultural production, but also had an inhibitory effect on it. This negative effect may stem from the excessive use of pesticides and chemical fertilizers, which leads to soil degradation, loss of biodiversity, and weakened carbon sink capacity, thereby hindering the synergistic development of ACSER and FS.

The proportion of arable land area (lnL) also shows a negative impact, which to some extent reflects the problem of poor quality of arable land utilization and serious diminishing marginal output in some areas of China, i.e., an increase in the area of arable land may not necessarily bring about an increase in the level of synergies, and on the contrary, it may lead to the wastage of resources and an increase in the burden on the environment. The effect of grain yield level (lnY) on the coupling synergy is −0.062 and significantly negative, indicating that in less developed agricultural areas, yield improvement often depends on high intensity inputs, which negatively affects CSER, which is consistent with the results of ‘high yield ≠ low carbon’ in recent years, suggesting the need to shift toward a green high-yield path grounded in ecological sustainability. However, food possession per capita (lnFP), despite a positive coefficient (0.024), did not pass the test of significance, suggesting that food possession per capita has limited synergistic effects on the two, which may be related to the mediating effects of factors such as food distribution efficiency, storage and transportation systems, and food wastage.

**Fig. 6:**
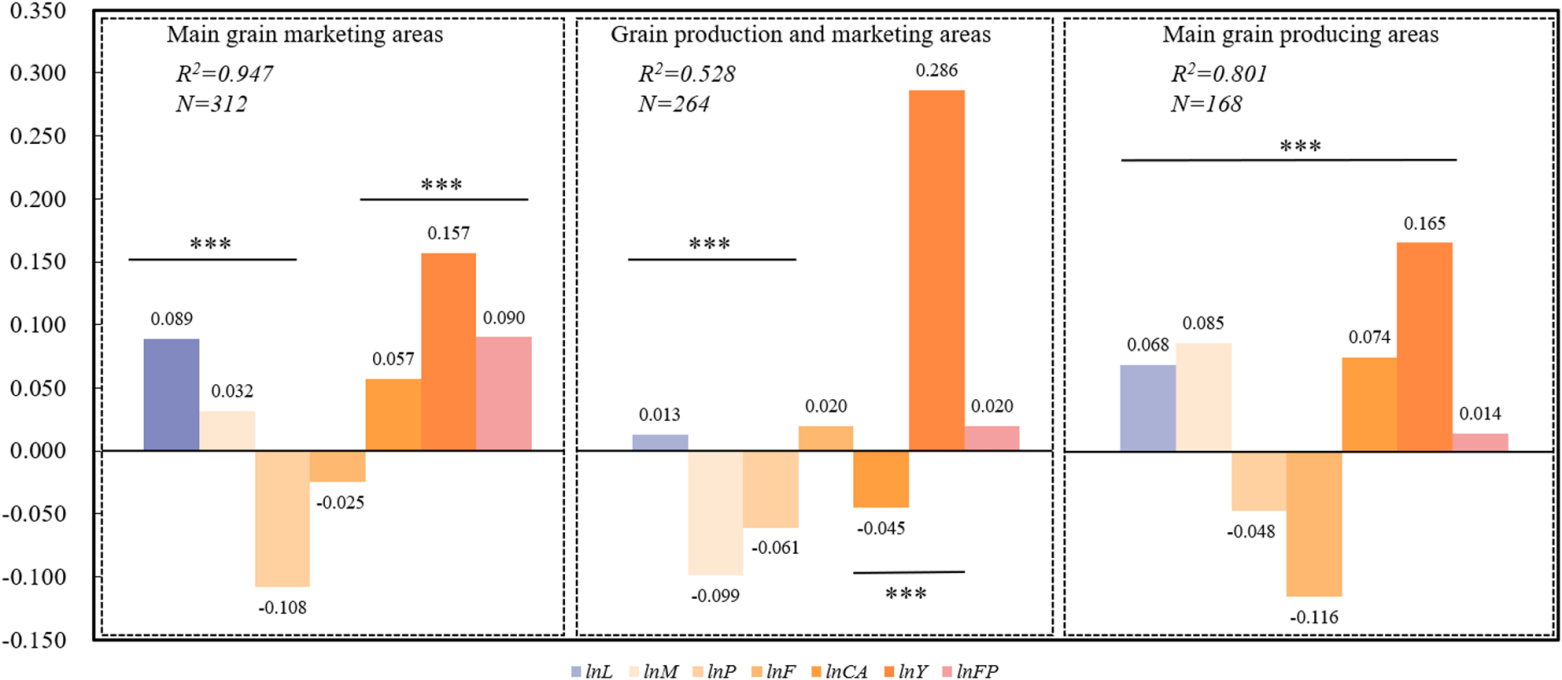
Estimation Results of Spatial SDM for CCD of Different Food Functional Areas

Figure 6 presents the regression estimation results of the coupled synergies of ACSER and FS in different food functional areas from the perspective of regional heterogeneity. By comparing the regression coefficients and their significance levels in each region, it is possible to clearly identify the variability of different regions in terms of agricultural resource utilization efficiency, input structure, and carbon emission reduction efficiency, which in turn provides a basis for regional precision policymaking.

The results showed that the R² of goodness of fit was 0.947 and 0.801 for the main production and marketing areas, respectively, indicating that the explanatory variables had strong explanatory power for the coupled synergy of ACSER and FS, whereas the R² of the production and marketing area was only 0.528, indicating that the synergy of the two in this area was affected by the interactions of multiple factors and policy interventions. In terms of individual variables, the proportion of arable land area (lnL) has a positive effect in all three major food functional regions, with higher coefficients in the main production and marketing regions, suggesting that arable land resources have a supportive effect on the synergistic promotion of agricultural carbon sequestration and FS in these regions; In contrast, in the production and marketing areas, the increase in the proportion of cultivated land area has less impact on the system coupling synergy, which may be influenced by factors such as urban expansion, land fragmentation or inefficient land use. The total power of agricultural machinery (lnM) shows a significant positive impact in the main production and marketing areas, indicating that agricultural mechanization has a positive role in promoting the synergistic development of ACSER and FS in agricultural cultivation, but in the production and marketing areas, agricultural mechanization is significantly negative, indicating that there may be a mismatch between machinery inputs and land and labor in the area, and thus synergistic benefits have not yet been formed. Pesticide application (lnP) was significantly negative in all regions, especially in the main production areas, where the negative effect was most prominent, indicating that high input pesticide use has inhibited the synergistic effect of the two, and that controlling the use of pesticides has become very urgent. Fertilizer application (lnF) exhibits a significantly negative effect only in the main sales regions, suggesting that areas with weaker environmental carrying capacities are more sensitive to fertilizer usage. In contrast, the main production and production-marketing areas do not show significant effects, which may be attributed to variations in fertilization practices and crop cultivation structures. CO₂ uptake by major crops (lnCA) shows a positive effect in both the main production and main sales regions, indicating that enhancing nutrient uptake efficiency is a key pathway for achieving the synergy between agricultural carbon sequestration and FS. However, it exhibits a significantly negative effect in the production-marketing areas, reflecting issues such as improper fertilization or resource inefficiency in these areas. Grain yield per unit area (lnY) demonstrates a significantly positive effect across all three major grain functional areas, with the most pronounced impact observed in the production-marketing areas. This indicates that yield remains a key driver of the synergy between FS and emission reduction, particularly in areas where land resources are scarce. The per capita food possession (lnFP) is significantly positive only in the main production areas, showing that it still reflects the synergistic situation of the two in a stable way in the region, while in the production and marketing areas and the main marketing areas, it may be less effective due to the effects of external perturbations, such as demographics. Overall, there are significant differences in the synergistic mechanisms of carbon sequestration in agricultural cultivation and FS in each food functional area, and the realization of synergistic optimization requires the formulation of policies tailored to regional characteristics to enhance the efficiency of resource allocation and the resilience of the system.

### Decomposition of spatial effects of coupled synergies of different food functional areas in China

The results of SDM effect decomposition showed (Table 6) that the spatial effect of ACSER and FS synergies of agricultural cultivation showed multidimensional characteristics. The direct effect of the proportion of cultivated land area (lnL) was − 0.149, the indirect effect was − 0.487, and the total effect amounted to −0.636, and all of them passed the 1% significance level test, indicating that the expansion of cultivated land not only inhibits the coordinated development of the two in this region, but also has a neighboring region It has a significant negative spillover effect, which may be related to the rough utilization of farmland and regional ecological pressure transmission, implying that the management of arable land should be shifted from quantity protection to quality improvement [44]. The total power of agricultural machinery (lnM) has a positive promotion effect on the synergy between the two in this region, with a direct effect of 0.017, but it transmits a negative effect in space, with an indirect effect of −0.060, causing the total effect to turn negative (−0.044). This indicates that there are problems such as resource competition and uneven allocation in the diffusion of agricultural mechanization between regions during the study period, so it is necessary to strengthen regional synergy and the construction of institutional support. The amount of pesticide application (lnP) showed a significant negative effect in this and neighboring regions, with a total effect of −0.186, indicating that excessive pesticide inputs inhibited the coordinated development of ACSER and FS, corroborating the necessity of green control and reduction of pesticide application. The direct effect of fertilizer application (lnF) was − 0.059, and the indirect effect was 0.198, leading to an overall effect of 0.139, indicating that there is a positive spillover effect of moderate fertilizer application in this region on food production and CSER in neighboring regions, but the precision of fertilizer application needs to be improved. CO₂ uptake by major crops (lnCA) was significantly positive in different food functional areas, which is a key driving factor to promote the synergistic development of CSER and FS, indicating again that improving nutrient uptake efficiency is an important way to promote the quality and efficiency of agriculture and guarantee FS. Comparatively, the level of food yields (lnY) harms mutual development (Table 6), suggesting that high yields may pose ecological risks due to high inputs and consumption, implying that enhancing ecological resilience becomes a necessity. The per capita food possession (lnFP) shows a positive effect in both the region and the neighboring regions, indicating that the rational allocation and sharing of food resources have a positive effect on improving the level of mutual development. Overall, crop CO₂ uptake efficiency and precision of agricultural inputs play a key role in the synergistic development of ACSER and FS.

**Table 6:** Spatial effect decomposition of CCD in different grain functional areas in China

	Variable	National level	Major grain producing areas	Grain production and marketing areas	Major grain marketing areas
Direct Effect	*lnL*	−0.149***(−5.38)	−0.038(−0.62)	−0.129***(−3.80)	0.100***(13.12)
	*lnM*	0.017***(−2.85)	−0.002(−0.34)	−0.012(−1.39)	0.061***(9.00)
	*lnP*	−0.065***(−8.59)	−0.064***(−5.49)	0.009(1.11)	−0.059***(−5.07)
	*lnF*	−0.059***(−4.90)	−0.132***(−7.55)	−0.057***(−3.81)	−0.083***(−4.54)
	*lnCA*	0.304***(−14.48)	0.198***(5.63)	0.207***(8.98)	0.017(0.96)
	*lnY*	−0.076***(−2.73)	0.012(0.21)	−0.047(−1.23)	0.209***(12.75)
	*lnFP*	0.036**(−2.11)	0.058(1.43)	0.096***(3.22)	0.032***(3.05)
Indirect Effect	*lnL*	−0.487***(−7.32)	0.060(0.61)	−0.295***(−6.27)	0.079***(3.93)
	*lnM*	−0.060***(−5.18)	0.017*(1.65)	−0.042***(−3.49)	−0.008(−0.94)
	*lnP*	−0.122***(−4.83)	−0.028(−1.53)	−0.023*(−1.76)	−0.018(−0.88)
	*lnF*	0.198***(−4.64)	0.021(0.70)	−0.026(−1.13)	0.046**(2.16)
	*lnCA*	0.127***(−2.95)	0.029(0.66)	0.012(0.33)	−0.021*(−1.68)
	*lnY*	−0.202***(−3.32)	0.051(0.55)	−0.161***(−3.01)	0.094***(5.07)
	*lnFP*	0.153***(−3.61)	−0.058(−0.88)	0.060*(1.65)	−0.066***(−3.74)
Total Effect	*lnL*	−0.636***(−8.93)	0.022(0.34)	−0.424***(−8.53)	0.179***(8.05)
	*lnM*	−0.044***(−3.81)	0.015(1.64)	−0.053***(−5.09)	0.052***(4.21)
	*lnP*	−0.186***(−6.24)	−0.092***(−4.85)	−0.014(−0.81)	−0.076**(−2.54)
	*lnF*	0.139***(−2.79)	−0.110***(−3.15)	−0.084***(−2.88)	−0.037(−1.04)
	*lnCA*	0.431***(−8.94)	0.227***(7.04)	0.219***(5.35)	−0.004(−0.15)
	*lnY*	−0.278***(−4.26)	0.062(1.03)	−0.208***(−3.83)	0.303***(12.94)
	*lnFP*	0.188***(−4.42)	0.001(0.02)	0.156***(3.57)	−0.033(−1.22)

***, **, and * denote significance at the 1%, 5%, and 10% levels, respectively. Values in parentheses are t-statistics

From a regional perspective, the main grain-producing areas exhibit notable differences between direct and indirect effects. The direct effects of the proportion of cultivated land area (lnL) and the total power of agricultural machinery (lnM) are not significant, indicating that changes in cultivated land area and total power of machinery do not play a prominent role in synergistic promotion of ACSER and FS in the region; while pesticide and fertilizer application (lnP and lnF) show significant negative direct effects, indicating that high-intensity agricultural inputs are an obstacle to the synergistic development of the region, especially fertilizer (−0.132***); CO₂ uptake by major crops (lnCA) shows a significant positive direct effect, indicating that enhancing crop CO₂ uptake efficiency is a key factor in promoting coordinated development. In addition, the level of grain yield (lnY) and per capita food possession (lnFP) did not show a significant effect in the main production areas, indicating that the driving effect of yield improvement and food resource allocation on mutual development is insufficient. Overall, the synergistic development of the main production areas relies more on the improvement of agro-ecological efficiency than on the expansion of a single production factor.

In the grain production-marketing areas, the coupled synergies of ACSER and FS show more complex and significant spatial linkages. The proportion of cultivated land area (lnL) showed both negative and significant direct (−0.129) and indirect (−0.295) effects, indicating that the crude cultivated land expansion pattern inhibited the synergistic development of both the region and the neighboring districts. The indirect effect of the total power of agricultural machinery is significantly negative (−0.042), reflecting a resource mismatch in the diffusion of mechanization across regions. Both pesticide and fertilizer inputs (lnP and lnF) exhibit significant negative direct effects, consistently reinforcing the notion that high agricultural input intensity undermines the coordinated development of CSER and FS. CO₂ uptake by major crops (lnCA), on the other hand, shows a strong positive direct effect (0.207***) and is prominent among the dominant factors. The level of grain yield (lnY) has a significant negative indirect effect (−0.161), indicating that the high-yield-dependent production mode has an inhibitory effect on the synergistic development of neighboring regions. Therefore, production and marketing areas need to promote the optimization and transformation of agricultural input methods based on strengthening green technology and resource allocation.

In the main grain marketing areas, both the proportion of cultivated land area (lnL) and the power of agricultural machinery (lnM) showed significant positive direct effects on the synergistic impacts of ACSER and FS (Table 6), indicating that agricultural infrastructure inputs are the key support to promote synergistic development in areas where agricultural resources are relatively scarce. Pesticides and fertilizers (lnP, lnF) constituted a significant inhibition of mutual development, where the indirect effect of fertilizer application was significantly positive (0.046, *p* < 0.05), suggesting that moderate fertilizer application in neighboring areas generates spillover benefits. The level of grain yield (lnY) is the core promoter of the main marketing area, and the direct effect (0.209) and indirect effect (0.094) are both highly significant, highlighting the importance of yield improvement in guaranteeing FS. The per capita food possession (lnFP) exhibits a clear “opposite-direction effect,” which may stem from the complexity of the grain circulation structure and fluctuations in demand in major sales regions.

### Robustness tests

To test the robustness of the results, this study replaces the baseline economic–geographic weight matrix with a pure geographic adjacency matrix, re-estimates the SDM model results, and decomposes the direct–indirect–total effects. Tables 7 and 8 present the re-evaluation results, which show that the signs of some variables have changed. This discrepancy primarily stems from differences in spatial weighting settings. However, overall, the direction and significance of core variables remain largely consistent- the proportion of ar-able land area, pesticide ap-plication, fertilizer application use continue to significantly inhibit mutual development; CO₂ uptake by major Crops remains a significant driver; and total power of agricultural machinery exerts a positive effect in primary production areas while transmitting negative effects in production-and-consumption areas. Spatial spillover effects showed slight convergence compared to the baseline setting, indicating stronger transmission of economic linkages under the economic-geographic weight matrix. Pure geographic adjacency better captures diffusion driven by “natural proximity,” demonstrating the SDM model’s high robustness.

**Table 7:** SDM Estimation results of CCD in different grain functional areas in China (Robustness Test)

Variable	National level	Major grain producing areas	Grain production and marketing areas	Major grain marketing areas
*lnL*	−0.109*** (−5.10)	0.084*** (3.10)	0.019** (2.03)	0.071*** (6.80)
*lnM*	0.021*** (3.65)	0.029*** (3.84)	−0.093*** (−6.55)	0.081*** (8.12)
*lnP*	−0.059*** (−8.90)	−0.102*** (−12.1)	−0.055*** (−4.97)	−0.047*** (−4.60)
*lnF*	−0.070*** (−4.85)	−0.024 (−1.26)	0.015 (1.21)	−0.103*** (−5.38)
*lnCA*	0.288*** (13.0)	0.062*** (4.12)	−0.038*** (−3.46)	0.069*** (2.70)
*lnY*	−0.060** (−2.40)	0.150*** (11.2)	0.271*** (12.1)	0.159*** (7.90)
*lnFP*	0.022*** (2.90)	0.017 (1.27)	0.010 (1.08)	0.013 (0.99)
ρ	0.418***	0.372***	0.401***	0.455***
R²	0.330	0.524	0.523	0.901

***, **, and * denote significance at the 1%, 5%, and 10% levels, respectively. Values in parentheses are t-statistics

**Table 8:** Spatial effect decomposition of CCD in different grain functional areas in China(Robustness Test)

	Variable	National level	Major grain producing areas	Grain production and marketing areas	Major grain marketing areas
Direct Effect	*lnL*	−0.141*** (−5.20)	−0.031 (−0.62)	−0.122*** (−3.86)	0.095*** (12.8)
	*lnM*	0.016*** (2.92)	−0.004 (−0.36)	−0.011 (−1.32)	0.058*** (8.85)
	*lnP*	−0.061*** (−8.10)	−0.061*** (−5.42)	0.008 (1.05)	−0.053*** (−5.09)
	*lnF*	−0.053** (−4.20)	−0.126*** (−7.41)	−0.055*** (−3.72)	−0.079*** (−4.60)
	*lnCA*	0.297*** (14.1)	0.191*** (5.51)	0.201*** (8.30)	0.016 (0.93)
	*lnY*	−0.071** (−2.55)	0.018 (0.23)	−0.021 (−1.21)	0.203*** (12.5)
	*lnFP*	0.033** (2.20)	0.054 (1.39)	0.091*** (3.05)	0.030** (2.95)
Indirect Effect	*lnL*	−0.452*** (−7.10)	0.058 (0.58)	−0.295*** (−6.05)	0.079*** (3.96)
	*lnM*	−0.057*** (−5.60)	0.016* (1.65)	−0.042** (−2.36)	−0.008 (−0.94)
	*lnP*	−0.115*** (−4.70)	−0.026 (−1.49)	−0.016 (−0.79)	−0.016 (−0.88)
	*lnF*	0.184*** (4.55)	0.019 (0.70)	0.018 (1.20)	0.046** (2.28)
	*lnCA*	0.119** (2.60)	0.028 (0.66)	0.015 (0.44)	−0.021 (−1.68)
	*lnY*	−0.188*** (−3.35)	−0.048 (−0.96)	−0.087** (−2.24)	0.012 (0.67)
	*lnFP*	0.141*** (3.60)	−0.056 (−0.85)	0.058* (1.74)	0.126*** (3.74)
Total Effect	*lnL*	−0.593*** (−8.60)	0.027 (0.31)	−0.417*** (−8.12)	0.174*** (8.05)
	*lnM*	−0.041*** (−3.85)	0.012 (1.13)	−0.053** (−2.51)	0.050*** (3.92)
	*lnP*	−0.176*** (−6.10)	−0.087*** (−4.81)	−0.008 (−0.41)	−0.069*** (−4.17)
	*lnF*	0.131** (2.78)	−0.107*** (−3.18)	−0.037** (−2.08)	0.05511(1.54)
	*lnCA*	0.416*** (8.95)	0.219*** (3.54)	0.216*** (3.29)	−0.005 (−0.31)
	*lnY*	−0.259*** (−4.26)	−0.030 (−0.59)	−0.108** (−2.46)	0.215*** (9.24)
	*lnFP*	0.174*** (4.42)	−0.002 (−0.03)	0.149*** (3.58)	0.156*** (3.98)

***, **, and * denote significance at the 1%, 5%, and 10% levels, respectively. Values in parentheses are t-statistics

## Discussion

### Spatial correlation characteristics

In the coupled synergistic study of ACSER and FS, spatial correlation characteristics reveal the dynamic process of structural differences in regional development. Among them, the global Moran’s I increased from 0.086 in 2000 to 0.337 in 2023, indicating that the coupled synergy of ACSER and FS in agricultural cultivation was spatially significantly positively correlated, and the correlation was increasing. This reflects that national agricultural green transformation and policy optimization promote the convergence of synergistic paths between regions [45, 46]. Although Moran’s I values in the main production and production-marketing areas shifted from negative to positive-indicating strengthened spatial clustering, most years did not reach statistical significance, suggesting the presence of structural imbalances and disparities in resource endowments within these functional areas. In the main marketing area, Moran’s I decreased continuously from 0.576 in 2000 to −0.206 in 2023, indicating that the coupling synergy in this area is spatially dispersed, and that rapid urbanization and scarcity of arable land resources are the main reasons affecting the synergistic development of ACSER and FS [47]. Localized Moran’s I further reveals the staged variation of regional agglomeration. Overall, the coupling synergy of ACSER and FS in China is characterized by a gradient of ‘high in the north and low in the south’, with the ‘high - high’ concentration remaining stable in the northern region due to the strong agricultural foundation and early promotion of green transformation. The South, on the other hand, has long been in a ‘low-low’ or ‘high-low’ situation, and the problem of synergistic development is more prominent. Particularly in 2015, the spatial pattern diverged significantly, and the synergistic capacity of some of the main producing provinces weakened, indicating that synergistic development does not advance linearly, but is influenced by a combination of multiple external perturbations and endogenous dynamics. By 2023, the Beijing-Tianjin-Hebei and Yellow-Huaihai regions will once again form stable ‘high-high’ agglomerations, but the ‘high-low’ and ‘low-low’ phenomena in the central and southwestern regions suggest that there is still a fault line in mutual development. So, it is necessary to strengthen the synergistic docking and optimization of factor reconstruction between regions [24]. Overall, the CCD for ACSER and FS in China has a spatial evolutionary trend from dispersion to agglomeration and from disorder to order. The spatial evolution pattern reveals a typical trajectory of “dual enhancement in environmental improvement and production efficiency.” The synergistic effects of policy, technology, and market mechanisms have enabled the agricultural system to gradually transition from “resource-consumption-driven” to “ecological efficiency-driven.” This mirrors the logic of the EU’s Common Agricultural Policy in promoting “green agriculture”: achieving compatibility between carbon reduction and food security through institutional incentives and technological innovation. However, unlike the EU’s approach of relying on subsidies to promote low-carbon farming and ecological restoration, China’s path has been more dependent on policy guidance and regional structural optimization, exhibiting phased characteristics and significant regional heterogeneity. Therefore, to enhance the operability and effectiveness of regional coordination, institutional mechanisms should be established for cross-regional flows of carbon and ecological resources, alongside a linkage mechanism between ecological compensation and grain regulation. Regions bearing ecological protection responsibilities and restricting high-energy-consuming production should receive fiscal transfer payments or ecological credit compensation. Cooperation between primary production areas and primary consumption areas in carbon compensation and grain reserve management should be encouraged. Implement a differentiated regional strategy: strengthen green technology and agricultural machinery emission reduction policies in main grain production areas; optimize intensive land use and efficient irrigation systems in grain production and marketing areas; enhance urban agriculture and promote “carbon-neutral agricultural products” consumption in main grain marketing areas. This will establish a synergistic closed-loop system integrating industry, ecology, and markets, thereby advancing the coordinated upgrading of agricultural carbon sequestration, emission reduction, and food security.

#### Spatial spillover effects

The analysis of spatial spillover effects based on SDM found that the coupled synergy of ACSER and FS at the national level has obvious spatial spillover effects, and that there are positive interactions between neighboring regions in terms of technology diffusion, policy transmission, and market linkages. There is significant regional heterogeneity in the impact of factors on the synergistic development of different food functional areas. Among them, crop CO₂ uptake showed significant positive spatial spillover effects at the national level and in the three major food functional areas, indicating that enhancing crop CSER and FS through optimizing varietal structure and improving fertilizer application strategies is a key pathway to promote the synergistic development of ACSER and FS. This result is consistent with the findings of Huang et al. on the synergistic effects of high-efficiency crops and industrial structure on the enhancement of regional carbon sink capacity and agroecology, as well as the national strategic goal of ‘promoting the selection, breeding, and dissemination of high-carbon sink crops’ [48]. The proportion of arable land area is a positive influence factor in both the main production area and the main marketing area, reflecting that the arable land resource endowment is still an important factor influencing the level of coupling coordination. Especially in the main production area, high-quality arable land guarantees the stability of the agroecosystem and the carrying capacity of FS. In the main marketing area, limited arable land is required to make up for the resource constraints by improving the effectiveness of the unit area, forming an elastic space for coupling enhancement. The impact of the total power of agricultural machinery shows a clear regional differentiation. In the main production areas, the increase in the level of agricultural mechanization effectively reduces the intensity of carbon emissions per unit of arable land and improves production efficiency, thus significantly promoting coordinated development; however, it has produced adverse effects in grain production and marketing areas. The reasons may stem from the fact that agricultural machinery and high-quality farmland are often concentrated in main grain production areas, leading to resource crowding out and diminishing returns in mechanization promotion in surrounding regions. Second, policy spillover effects may also play a significant role. Cross-regional mobility and implementation disparities in agricultural machinery subsidies and related support policies lead to misallocation and inefficient utilization of machinery resources. Structural constraints further exacerbate this phenomenon. In main grain marketing areas, characterized by limited arable land and small-scale operations, exhibit low machinery utilization rates and limited gains in output efficiency per unit, creating negative spatial spillovers. Zhang et al. showed that there is an ‘over-investment-low utilization’ contradiction between machinery inputs and operational capacity in some provinces in the central and western production and marketing areas, which affects the release of the benefits of green agriculture, and this conclusion is mutually corroborated by the above results of this study [48]. However, compared with international experience, China’s spatial spillover mechanism bears similarities to the EU’s Carbon Farming Initiative while also exhibiting significant differences. The EU promotes low-carbon farming practices and bio-carbon sequestration measures among farmers through fiscal incentives and market-based carbon credit mechanisms, with spillover effects primarily diffusing via technical standards and subsidy systems. In contrast, China’s synergistic enhancement relies more heavily on policy transmission and inter-regional coordination, forming a diffusion pathway characterized by “policy guidance—market response—synergistic convergence.” In contrast, the U.S. “Precision Agriculture and Carbon Credit Mechanism” places greater emphasis on information technology and market signals, achieving economic-ecological balance in agricultural systems through carbon monitoring, blockchain traceability, and farm-level carbon credit trading. China’s approach is characterized by strong administrative leadership, high spatial dependency, and significant regional variations, making it more suited to a spatial regulation model grounded in a dual economic-geographic matrix. Pesticide application generally showed a significant negative effect nationwide, indicating that the high-input agricultural model caused significant suppression of the agroecosystem, which in turn constrained the synergistic optimization between the agricultural system and the food system. Zhang et al. found through empirical analysis of a panel data model that pesticide inputs present a double superimposed effect on agricultural carbon emissions and environmental pollution, which is an important obstacle affecting the process of green transformation of agriculture [49]. Therefore, in promoting ACSER, controlling the excessive use of pesticides and promoting green prevention and control technologies remain one of the key paths to enhance the ability to harmonize ACSER and FS. In addition, the impact of food yield level on coupling synergy is particularly prominent in the production and marketing areas, reflecting that high and stable yields are still the core support for the stable operation of the food system in the region. Although higher yields can help secure food supply and ease resource pressure, over-reliance on higher yields may also bring ecological risks such as fertilizer dependence and overdraft of arable land, thus reducing the long-term synergy between ACSER and FS.

### Direct versus indirect effects

The effect decomposition of SDM showed that the synergistic development of CSER from agricultural cultivation and food was not only directly affected by the agricultural resource inputs in the region, but also driven by significant spatial spillover effects, presenting a complex multidimensional spatial feature. At the national level, the significant negative total effect of the proportion of arable land area indicates that the expansion of arable land at the current stage is more often reflected in the crude utilization, which is not conducive to the synergistic development of the local area, but also exacerbates the ecological pressure on the neighboring areas, highlighting the urgent need to shift from quantitative growth to the quality enhancement of the development strategy [22]. In contrast, the EU’s Common Agricultural Policy (CAP) reforms have controlled disorderly farmland expansion through carbon subsidies and ecological constraints, enhancing both carbon sequestration and grain production efficiency per unit area. This experience offers valuable insights for China’s policy orientation toward transforming quantitative growth into qualitative improvement. Agricultural mechanization has a certain promotional effect locally, but its spatially transmitted negative effect suggests an uneven allocation of mechanical resources between regions and potential competition problems, which need to be strengthened in terms of institutional coordination and regional linkage governance. At the same time, pesticide and fertilizer application generally showed a significant negative effect, indicating that the high-input agricultural model in the local and neighboring regions constitute an inhibition of the synergistic development, especially the main producing areas and production and marketing areas are more obvious, thus confirming the necessity of the policy direction of green prevention and control and reduction of fertilizer application [50]. In contrast, crop CO₂ uptake showed a significant positive effect in all three major food functional areas, which is a key driver of synergistic development enhancement, suggesting that improving crop carbon uptake efficiency and carbon sink capacity is an effective pathway for realizing green efficiency in agriculture. The negative spatial effect of the level of grain yields indicates that the agricultural development model that relies on high yields has not brought about synergistic benefits in some regions, but rather has brought about ecological pressures due to high inputs and consumption, weakening the synergistic relationship between agriculture and FS, and that FS strategies should therefore be reshaped from the perspective of ecological resilience in the future. Regional analyses show that there are significant differences in the impact pathways of different food functional areas. The coupled synergistic development of the main production areas is dominated by the improvement of agro-ecological efficiency, and the negative impact of traditional input methods is obvious; the production and marketing areas are facing strong spatial linkage pressures, and need to promote the coordination of cross-regional mechanisms; while the main marketing areas are relying on the infrastructure and yields to drive synergistic levels of enhancement, but the complexity of the grain circulation system is still a constraining factor. Overall, differentiated governance strategies should be implemented across functional zones: promoting green agricultural machinery subsidies and low-carbon farming practices in the main production areas; refining carbon footprint accounting and sustainable development in the production and marketing areas; and establishing market-oriented mechanisms for “low-carbon agricultural products” in the main marketing areas, thereby using market demand to drive green transformation at the production end. This approach will foster synergistic and efficient collaboration between carbon sequestration through agricultural cultivation and food security, while advancing green transformation.

#### Research limitations

Although this paper systematically explores the spatiotemporal measurement and impact mechanisms of synergistic development between carbon sequestration and emission reduction in agricultural cropping and food security, certain limitations remain. First, constrained by data availability and consistency, it fails to fully reveal spatial heterogeneity at the county or agricultural district level. Future research could integrate remote sensing monitoring or high-resolution statistical data to conduct multi-scale comparative studies. Second, the variables selected for this study remain imperfect. For instance, factors such as farmland management practices, climate fluctuations, and policy interventions were not incorporated into the model, potentially introducing biases in the results. Finally, this study focuses on the overall characteristics of the national level and grain production functional zones. Future research could further integrate scenario simulations or system dynamics models to assess synergistic evolutionary trends under different policy pathways, thereby providing more actionable decision support for advancing agricultural low-carbon transformation and ensuring food security through zoned and categorized approaches.

## Conclusions

This study focuses on China’s food functional areas from 2000 to 2023. Through the modified CCD model, SDM, and regional heterogeneity analysis, it explores the spatial patterns and driving mechanisms of synergistic development at both national and functional-area scales. The results confirm a significant coupling and synergistic relationship between ACSER and FS, while extending the analytical paradigm beyond traditional static models. By establishing an analytical framework with both dynamic and spatial explanatory power, this study provides new theoretical insights and methodological innovations to promote the coordinated advancement of China’s agricultural “dual-carbon” objectives and food security strategies. The main conclusions are as follows:

1. The level of synergistic development of ACSER and FS in China as a whole shows a spatial gradient characterized by “high in the north and low in the south”, and exhibits significant spatial agglomeration. Among them, the spatial agglomeration of the main production areas and production and marketing areas has increased, but has not reached a significant level; while the main marketing areas have shown a continuous trend of spatial dispersion, reflecting the differences between functional areas in terms of resource endowment and the structure of agricultural inputs and outputs.
2. The results of SDM indicate that the CCD of ACSER and FS has a significant spatial spillover effect, suggesting that there is a linkage in the level of development between regions. Crop CO₂ uptake, as an important characterization of agricultural carbon sink capacity, is a key positive driver for coordinated development. In contrast, the proportion of arable land area and the intensity of pesticide and fertilizer application have significant negative effects on both local and neighboring regions, revealing that high-intensity agricultural inputs may impose constraints on regional agricultural sustainability.
3. The analysis of regional heterogeneity further reveals that there are significant differences in the paths and influence mechanisms of synergistic development in different food functional areas. In the main production area, the enhancement of crop CO₂ absorption effectively promotes the enhancement of CCD level; production and marketing areas are subject to resource mismatch and factor allocation imbalance, and the synergistic mechanism is more complicated; in the main marketing area, the total power of agricultural machinery has a significant role in promoting the local CCD, but the trans-regional conduction process produces a negative spillover effect, which reflects the imbalance in agricultural resource allocation and the urgent need for synergistic inter-regional governance.

In summary, the synergistic development of ACSER and food security is not only an important strategic issue in the context of realizing the goal of “dual-carbon”, but also related to the fundamental guarantee of national food security and sustainable development of agriculture. The research results of this paper provide a scientific basis for promoting the green and low-carbon development of agriculture and food security in different regions. In the future, inter-regional coordination and linkage should be strengthened to promote the green transformation of agricultural production methods and enhance the carbon sink capacity of the agricultural system while realizing higher-quality food security.

## Supplementary Information

Supplementary Material 1

## Data Availability

Data used in this paper come from open website and government, which has shown accessibility in this manuscript.

## Abbreviations

ACSER: Agricultural carbon sequestration and emission reduction
FS: Food security
CCD: Coupling coordination degree
SDM: Spatial durbin model
CSER: Carbon sequestration and emission reduction
GHG: Green house gas
L: Proportion of arable land area
M: Total power of agricultural machinery
P: Pesticide application
F: Fertilizer application
CA: CO₂ uptake by major crops
Y: Grain yield level
FP: Per capita food possession
LM: The spatial lagrangian
LR: The likelihood ratio
CCS: Crop-based carbon sequestration
