# Supplementary figures and images for: Spatial effects of the synergistic development between agricultural carbon sequestration and emission reduction and food security across China’s grain functional areas
Source: Carbon Balance Manag. 2025 Dec 12;21:2. doi: 10.1186/s13021-025-00369-2 (PMC12771897; doi:10.1186/s13021-025-00369-2)

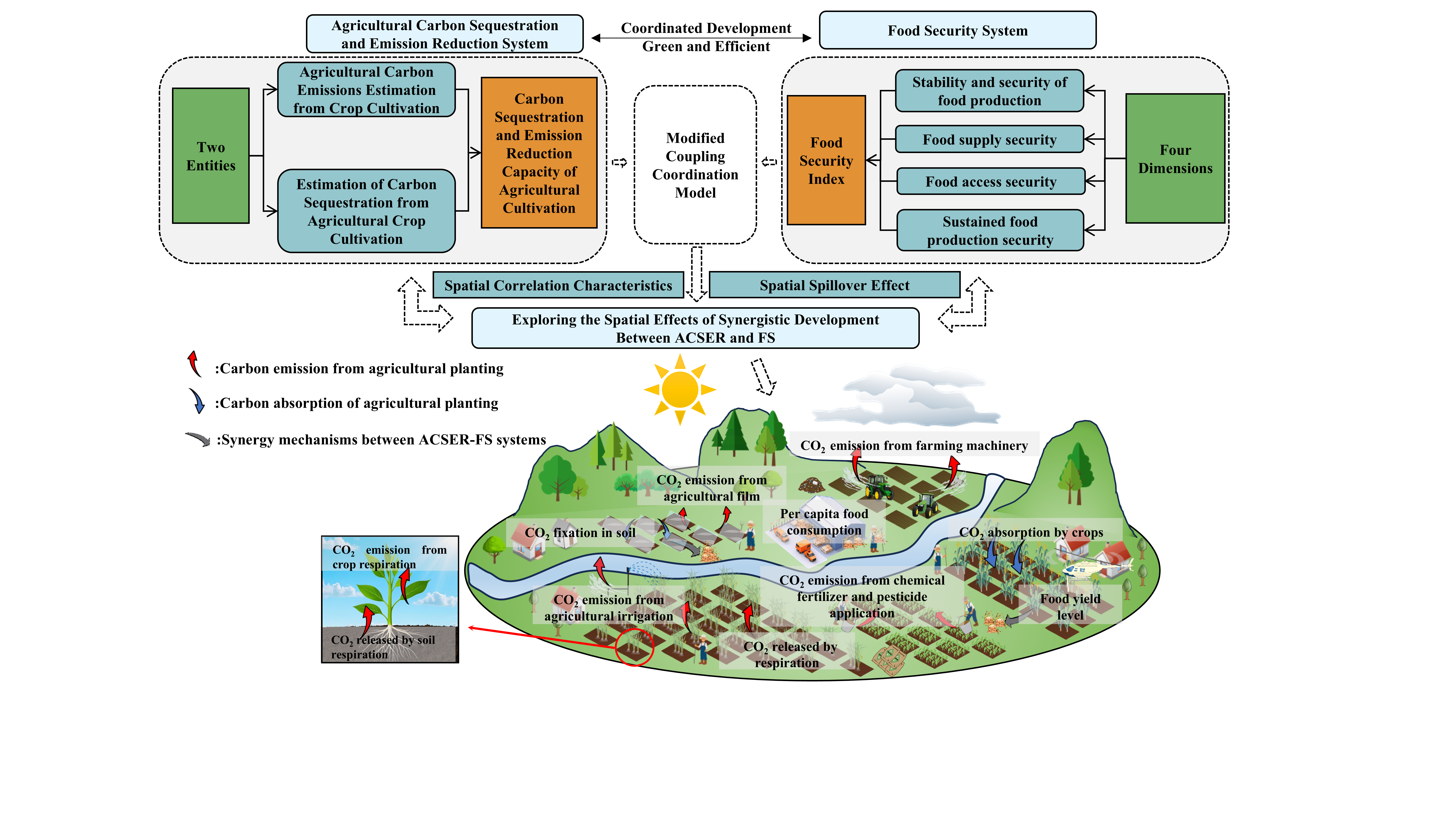

Supplement: Supplementary file 1 — Supplementary Material 1 [file 13021_2025_369_MOESM1_ESM.png]
